# Characterization of the Subchondral Bone and Pain Behavior Changes in a Novel Bipedal Standing Mouse Model of Facet Joint Osteoarthritis

**DOI:** 10.1155/2020/8861347

**Published:** 2020-11-09

**Authors:** Miao Li, Wen-qing Xie, Miao He, Deng-jie Yu, Da-qi Xu, Wen-feng Xiao, Yong Cao

**Affiliations:** ^1^Department of Spine Surgery and Orthopaedics, Xiangya Hospital, Central South University, 410008 Changsha, China; ^2^Key Laboratory of Organ Injury, Aging and Regenerative Medicine of Hunan Province, 410008 Changsha, China; ^3^Department of Orthopaedics, Xiangya Hospital, Central South University, 410008 Changsha, China; ^4^Department of Sports Medicine, Xiangya Hospital, Central South University, 410008 Changsha, China; ^5^National Clinical Research Center for Geriatric Disorders, Xiangya Hospital, Central South University, 410008 Changsha, China

## Abstract

**Background:**

The subchondral bone parallels with the progression of osteoarthritis (OA). However, the biomechanical properties and histopathological changes of subchondral bone changes in the lumbar facet joint (LFJ) after long-term axial loading on the spine have not been explored. In this study, we aimed to investigate the subchondral bone histopathological changes that occur in the LFJ and pain behaviors in a novel bipedal standing mouse model.

**Methods:**

Sixteen 8-week-old male C57BL/6 mice were randomly assigned into bipedal standing and control groups. A finite element stimulate model based on the micro-CT data was generated to simulate the von Mises stress distribution on the LFJ during different positions. The spine pain behaviors tests were analysis. In addition, the change in the subchondral bone of the LFJ was assessed by histological and immunohistochemistry staining.

**Results:**

The computerized simulation of the von Mises stress distribution in the superior articular process of LFJ at the spine level 5 in the lying position increased and reached a maximum value at the bipedal standing posture. The spine pain behavior test revealed that the threshold of pressure tolerance decreased significantly in bipedal groups relative to control groups, whereas the mechanical hyperalgesia of the hind paw increased significantly in bipedal groups relative to control groups. The axial load accelerates LFJ degeneration with increased histological scores in bipedal groups. The expression of type II collagen and aggrecan (ACAN) was significantly decreased in the bipedal groups compared with the control groups, whereas the expression of MMP13 was increased. Compared with the control groups, the osteoclast activity was activated with higher TRAP-positive staining and associated with increased CD-31-positive vessels and GCRP-positive nerve ending expression in the subchondral bone of LFJ.

**Conclusion:**

Collectively, long-term axial loading induces the development of spine hyperalgesia in mice associate with increased osteoclast activity and aberrant angiogenesis and nerve invasion into the subchondral bone of LFJ that stimulates the natural pathological change in human LFJ OA. These results indicate that aberrant bone remodeling associate with aberrant nerve innervation in the subchondral bone has a potential as a therapeutic target in LFJ OA pain.

## 1. Background

Lumbar facet joint (LFJ) osteoarthritis (OA) is implicated as an important cause of low back pain, which in turn places an enormous burden on the social health-care system [1, 2]. The facet joint has a similar characteristic as those of other synovial joints, such as the knee, and plays an important role in load transmission of the spine. However, the facet joint OA has to date received far less critical investigation than knee OA [1]. The fact that pain originating from the LFJ is a common cause of low back pain, and that the prevalence of LFJ OA pain has been estimated to range from 7 to 75% among the elderly population reporting low back pain [1, 3–5].

The LFJ is a true synovial joint composed of the articular cartilage covering the surfaces of each of facts, a thickened layer of subchondral bone, a synovium, and an articular capsule [1, 6]. The LFJ OA is viewed as an organ disease that affects the entire facet joints and is characterized pathologically by focal loss of the articular cartilage associated with subchondral bone change, varying degrees of osteophyte formation and synovitis [1, 7, 8].

Pain from the LFJ probably derives from multiple tissues of the facet joint [9]. A recent study revealed that their capsule tissue is well innervated by the free nerve during degeneration [1, 10]. The mechanoreceptors and upregulated inflammatory cytokines have also been identified in the facet joint capsular tissue in degenerative disc disease that could be the source of pain [9]. Recently, increasing evidence suggests that the contribution of the subchondral bone to the physiopathology of OA is of great interest.

The subchondral bone is a shock absorber in weight-bearing joints and plays a crucial role in the initiation and progression of OA [11]. It is now recognized that the subchondral bone is responsible for peripheral neuronal sensitization and can result in normal activities, causing pain. In OA conditions, inflammation and sensory nerve growth have been noted to coexist in the subchondral bone, indicating that it could be an important source of pain in OA [9]. As subchondral bone abnormalities appear in OA, this may be the target that leads to novel approaches for the development of OA pain treatment.

Biomechanical testing of isolated spinal segments has demonstrated that up to 33% of the total axial load of the spine segment can be borne by facet joints [12, 13]. Excessive mechanical loading can contribute to the initiation of spine degeneration [12, 14]. A previous investigation also found great variation in intradiscal pressure (IDP) when the sheep were standing from the lying position and 5- to 6-fold greater than the IDP recorded in the lying position [15, 16]. It is highly needed for the development of an animal model of noninvasive cumulative axial loading on the spine by making the animal maintain an upright posture to mimic the processes of degeneration in humans. Ao and Wang constructed a novel bipedal standing mouse model by placing them in limited water to induce the bipedal posture for a long period of time that can simulate the pathogenesis of spinal degeneration caused by increased axial load stress [17].

This model successfully reproduced LFJ degeneration; however, the 3D microstructure and histopathology of the subchondral bone change in osteoarthritic facet joints have not been extensively explored. In this study, we aimed to investigate the subchondral bone microstructure and histopathological features that occur in facet joints, and the pain behaviors change obtained from a bipedal standing mouse model.

## 2. Methods

### 2.1. Experimental Animals

All animal procedures in this study were conducted with the approval of the Animal Ethics Committee of the Xiangya hospital of Central South University (protocol number: 2019N0106). Sixteen C57BL/6 mice (8 weeks old) were purchased from the Animal Center of Central South University (Changsha, China) and randomly divided into two groups of eight mice each, the normal control and experimental groups. In the experimental groups, the mice were placed in a beaker containing limited water to induce the bipedal standing posture according to a previously described protocol. The mice in the control group were placed in the same chamber without the added water. These mice in the two groups underwent two different interventions for a total of 6 hours each day and were free to access food and water. Six months after the intervention, all mice were anesthetized with an intraperitoneal injection of 5% ketamine hydrochloride plus 0.5% diazepam following the standard protocol. All lumbar spines were harvested and fixed in 10% buffered formalin for micro-CT scanning, finite element analysis, and histopathological analysis. At the schedule timepoint, the mice was placed in a chamber with a prolonged exposure (more than ten minutes) in the CO_2_ monitoring continuously until the mice are no longer moving.

### 2.2. Micro-CT Analysis of the LFJ Subchondral Bone

The fixed lumbar spines from L1 to L5 were captured by a micro-CT scanner (Skyscan 1076, Skyscan, Antwerp, Belgium) with an isotropic voxel size of 6 *μ*m. The X-ray tube voltage was 80 kV, and the current was 100 *μ*A with a 0.5 mm aluminum filter. NRecon and CTVol software was used for transverse 2D cross-sectional reconstructions and 3D image visualization. For the quantitative analysis of the subchondral bone, the parameters including bone volume fraction, which describes the ratio of bone volume over tissue volume (BV/TV, %), three-dimensional trabecular bone thickness (TbTh), the ratio of the bone surface area to bone volume (BS/BV), the trabecular bone number (TB. N, mm), and the trabecular bone space (Tb. Sp, mm) were calculated.

### 2.3. Computerized Stimulation of the LFJ Stress Distribution

A finite element model of the mouse lumbar spine was developed as previously described with some modifications [18]. Micro-CT tomography images were acquired from the scanning mice. Simpleware (Simpleware, Ltd., Exeter, UK) was used for preprocessing and model reconstruction, and ABAQUS (6.10; Simulia Inc, Providence, Rhode Island, USA) was used for simulation. We developed a 3-dimensional, nonlinear FE model of the lumbar spine that consisted of an L4-5 LFJ using the finite element software Ansys version 1. In this study, facet joints were modeled using a frictionless surface-to-surface contact between zygopophysis with an average gap of 0.2 mm. The facet joint is subjected to the lying and standing position. Loading act on the facet joint must consider that the spine must support the whole-body weight at standing positions. Static analysis is conducted to measure the von Mises stress on the facet joint.

### 2.4. Histological and Pathological Assessment

The L4-5 LFJ was harvested and decalcified in 10% EDTA (pH 7.4) and embedded in paraffin. A 4-*μ*m-thick crossoriented section of the superior articular process of L5 was stained with safranin O and fast green (Sigma) to observe the morphology. A histological scoring system was used to characterize the features of the facet joints as previously described. Osteoclast activity was detected by tartrate-resistant acid phosphatase (TRAP) staining according to the standard protocol (Sigma-Aldrich). For immunohistology, slides were first incubated with antigen retrieval buffer (Abcam) and blocking buffer. Then, sections were incubated with anti-MMP13 (1 : 200, Abcam, Cambridge, MA), anti-type II collagen (1 : 200, Abcam, Cambridge, MA), anti-ACAN(1 : 200, Abcam, Cambridge, MA), anti-CD31 (1 : 200, Abcam, Cambridge, MA), and anti-CGRP (1 : 200, Abcam, Cambridge, MA) primary antibodies. For immunofluorescence, the sections were counterstained with 4′,6-diamidino-2-phenylindole (DAPI; Sigma). For immunohistochemistry, a horseradish peroxidase–streptavidin detection system (Dako, Agilent Technologies) was used to detect immunoactivity, followed by counterstaining with ethyl green (Sigma-Aldrich). Or the sections were then counterstained with hematoxylin (Dako). All the sections were observed under the microscope (Zeiss) and scored in a blinded fashion. Five fields of the whole subchondral bone area per specimen per mouse in each group were randomly selected for quantitative histomorphometry analysis.

### 2.5. Spine Pain Behavioral Assessment

Behavioral testing was performed between two groups before the mice were anesthetized for spine harvested. Vocalization thresholds in response to the force of an applied force gauge (SMALGO algometer; Bioseb) were measured to reflect the spine pain behaviors. Briefly, a sensor tip was directly pressed on the dorsal skin of the mice at the L4–L5 position. The pressure force was increased gradually at a constant speed (50 g/s) until an audible vocalization was heard. The curve of the pressure force was recorded by using BIO-CIS software (Bioseb). Two tests were performed by an observer who was blind to the study design, and the mean value was calculated as the nociceptive threshold. The hind paw withdrawal frequency of mice responding to a mechanical stimulus was determined using von Frey filaments (Stoelting, Wood Dale, IL). Mice were placed individually in acrylic cages with a mesh floor. Von Frey filaments were applied to the mid-plantar surface of the hind paw with enough pressure to buckle the filaments. If the mice withdraw, or shake the paw, it is considered to have had a positive response. Von Frey filament was used to apply physical stimulation at 1 s interval for ten times when the mouse hind paw contacts with the mesh. The force is increased manually until paw withdrawal occurs. At the meantime, the force was record. Mechanical withdrawal frequency was calculated as the percentage of withdrawal times in response to ten stimulations.

### 2.6. Statistical Analysis

All grouped data are presented as the means ± standarddeviations (SD) and analyzed by using SPSS, version 15.0, software (IBM Corp.). Two-tailed unpaired Student's *t* test was used to compare between two groups. The rest of the data were analyzed using either one-way or two-way ANOVA, with post hoc Tukey's multiple comparisons. A *p* value of ≤0.05 was considered significant.

## 3. Results

### 3.1. Von Mises Stress Property of Superior Articular Processes (SAPs) in the LFJ during Different Positions

**Figure 1:**
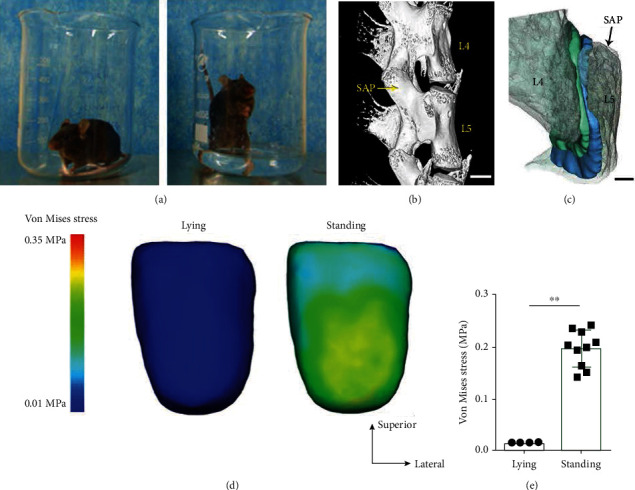
The bipedal standing mouse model and von Mises stress distribution on the surface of SAPs in LFJ.(a)The mice were placed in a beaker with or without limited water to induce the bipedal standing posture. (b) The 3D image of micro-CT scanning of the spine. (c)The finite element stimulation model of SAPs. (d) The von Mises stress distribution on the surface of SAPs during different positions and (e) quantitative analysis data. The data are presented as the mean ± SD. ^∗∗^*p* < 0.01 for differences between the control group and the bipedal standing group. Scalebar = 200*μ*m.

The mice were placed in a beaker containing limited water to induce the bipedal standing posture (Figure 1(a)). The L5 SAPs have been selected for our region of research interest (ROI) (Figure 1(b)). An established finite element model of the human LFJ was used to stimulate the stress distribution in the superior articular processes (SAPs) in different positions (Figures 1(b) and 1(c)). The simulation began from a lying position, and no contact between the two articular facets was scanned via micro-CT. Since the facet joint supports the physiologic motion of the spine, the articular surfaces remain in contact during the bipedal standing position. It was observed that the computerized simulation of the von Mises stress distribution increased steadily from approximately 0.01 ± 0.0014 MPa in the SAPs of the LFJ at level 5 in the lying position and reached a maximum value of 0.24 ± 0.0376 MPa at the bipedal standing posture (Figures 1(d) and 1(e)).

### 3.2. Development of Spinal Hypersensitivity in the Bipedal Standing Mice Model

**Figure 2:**
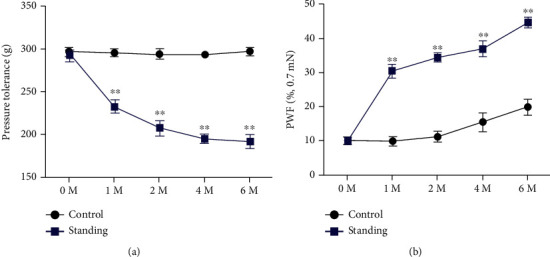
Symptomatic spinal pain behavior in the long-term bipedal standing mouse model. (a). Pressure hyperalgesia of the spine was measured as the force threshold to induce the vocalization by a force gauge. (b). The hind paw withdrawal frequency (PWF) responding to mechanical stimulation (von Frey, 0.7 mN). ^∗∗^*p* < 0.01 for differences between the control group and the bipedal standing group at the corresponding time points.

After six months of bipedal standing, the vocalization threshold in response to force applied on the mice spine L4/L5 level was measured. The results demonstrated that pressure tolerance decreased significantly at six months in bipedal standing groups relative to mice than in control groups (Figure 2(a)). In parallel, the von Frey test showed that the paw withdraw frequency increases significantly at 6 months in bipedal standing groups (Figure 2(b)). These results of spinal pain behavior tests indicating that that long-term standing will lead mice to develop spine hyperalgesia.

### 3.3. Pathologic Change in the Cartilage in the LFJ after Long Bipedal Standing Induction

**Figure 3:**
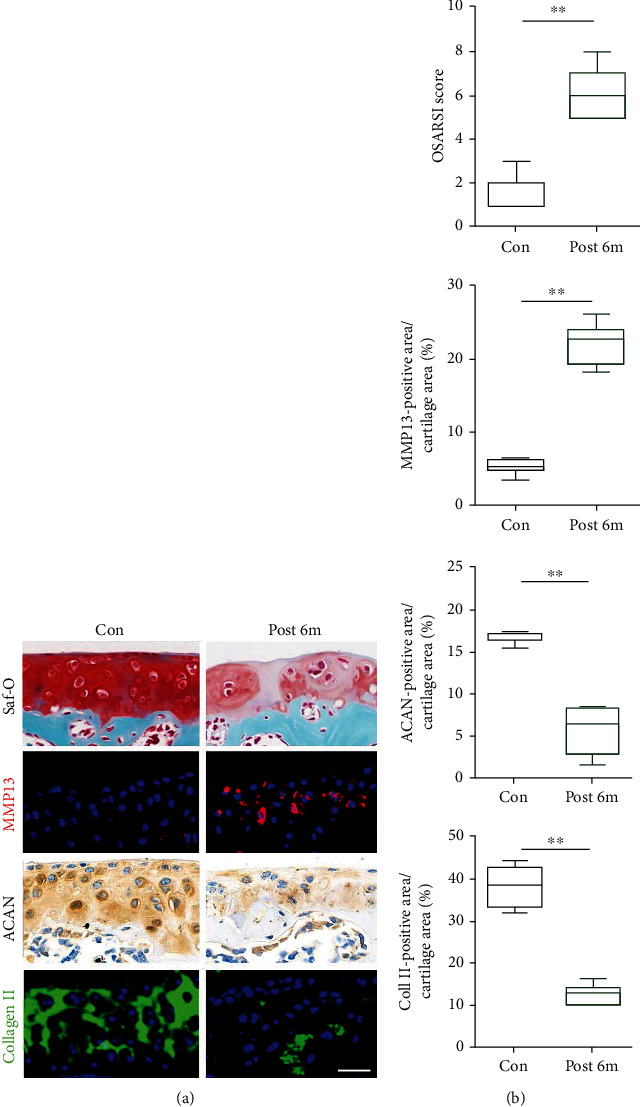
Axial loading acts on the spine to induce LFJ cartilage degradation. (a) Histological change in the LFJ with safranin O staining (upper). Changes in the expression of MMP13, ACAN (middle), and type II collagen (lower) in the LFJ cartilage with immunofluorescent and immunohistochemistry staining. (b) Quantitative analysis of LFJ cartilage degeneration in different groups and evaluation of MMP13-positive area, ACAN positive areas, and type II collagen-positive area in the LFJ cartilage. The data are presented as the mean ± SD. ^∗∗^*p* < 0.01 for differences between the control group and the bipedal standing group. Scalebar = 40*μ*m.

Cartilage is one of the key anatomical structures of LFJ, and joint degeneration always induces pathological changes in cartilage. As shown in Figure 3(a), the pathologic changes of the degenerated LFJ are clearly presented in the long period of time-bipedal standing mice. Staining with Saf-o revealed reduced cartilage layer thickness, with proteoglycan matrix depletion and chondrocyte loss in the SAPs after 6 months of exercise in bipedal standing groups (Figure 3(a)). To quantify the severity of cartilage degeneration, we evaluated the Osteoarthritis Research Society International (OARSI) scores of the SAPs between the control and the bipedal standing groups. OARSI scores revealed a dramatic increase in OARSI scores in the bipedal group compared to the control group (Figure 3(b)). Moreover, the percentages of MMP13-positive chondrocytes were significantly increased, indicating that a long period of bipedal standing induces LFJ cartilage degradation. In contrast, Col II and ACAN expression indicating a protective marker was significantly reduced in the bipedal groups (Figures 3(a) and 3(b)). Our findings reveal that the novel bipedal standing mouse model caused the normal architecture of the cartilage to be lost, leading to the successful development of LFJ osteoarthritis and consistent with previous reports.

### 3.4. 3D Morphological Change in the Subchondral Bone in Bipedal Standing-Induced LFJ OA

**Figure 4:**
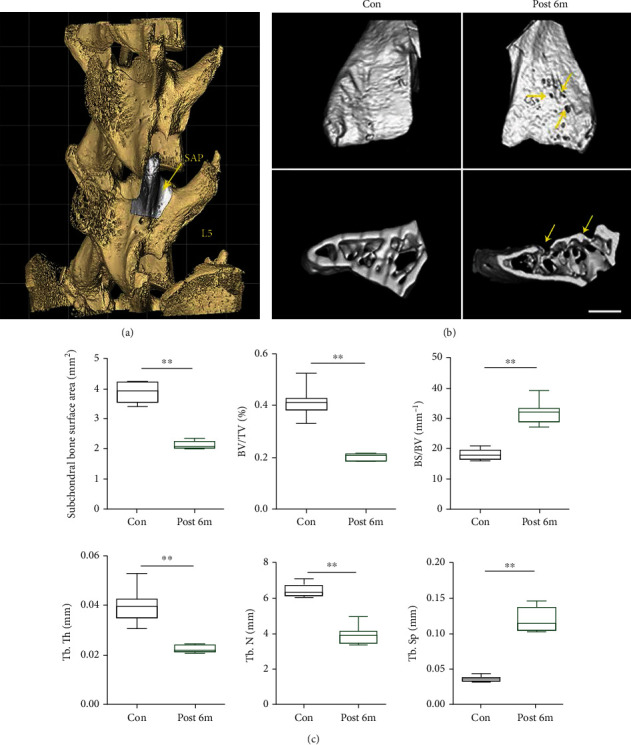
Axial loading led to the subchondral bone collapse. (a) The 3D image of SAP of lumbar 5. (b) 3D micro-CT image of the LFJ subchondral bone between control and bipedal standing mouse models. A series of subchondral bone cavities were visualized in a 6-month long bipedal standing mouse model. (c) Quantitative analysis of the morphological parameters of the subchondral bone change in different groups. The data are presented as the mean ± SD. ^∗∗^*p* < 0.01 for differences between the control group and the bipedal standing group. Scalebar = 200*μ*m.

Since less is known about the 3D morphological change in the subchondral bone in long-term bipedal standing-induced LFJ OA mice, we used micro-CT to visualize the microstructural changes in the subchondral bone of LFJ. As shown in Figure 4(a), the SAPs of L5 were selected; it demonstrated that the bipedal-induced LFJ OA led to collapse that was not limited to the cartilage, and the subchondral bone was also affected. The appearance of a localized cavity on the surface of the subchondral bone could be observed in the bipedal groups, whereas the surface in the control group was integrated (Figure 4(b)). Six months of long-term bipedal standing decreased the subchondral bone surface area in SAPs (bipedal standing groups 3.96 ± 0.034 mm^2^ vs. control groups 2.02 ± 0.063 mm^2^). The subchondral bone BV/TV ratio in LJF OA mice dramatically decreased relative to the control groups (bipedal standing groups 41.46% vs. control groups 21.98%). The Tb. Th and Tb. N of the subchondral bone significantly decreased with abnormal morphology, whereas the ratio of the bone surface area to bone volume and Tb. Sp increased after two months of bipedal standing (Figure 4(c)). The results reveal that altered mechanical loading in LFJ leads to accelerated subchondral bone remodeling and induced subchondral bone resorption.

### 3.5. Aberrant Nerve Invasion in the Subchondral Bone in Bipedal Standing-Induced LFJ OA

**Figure 5:**
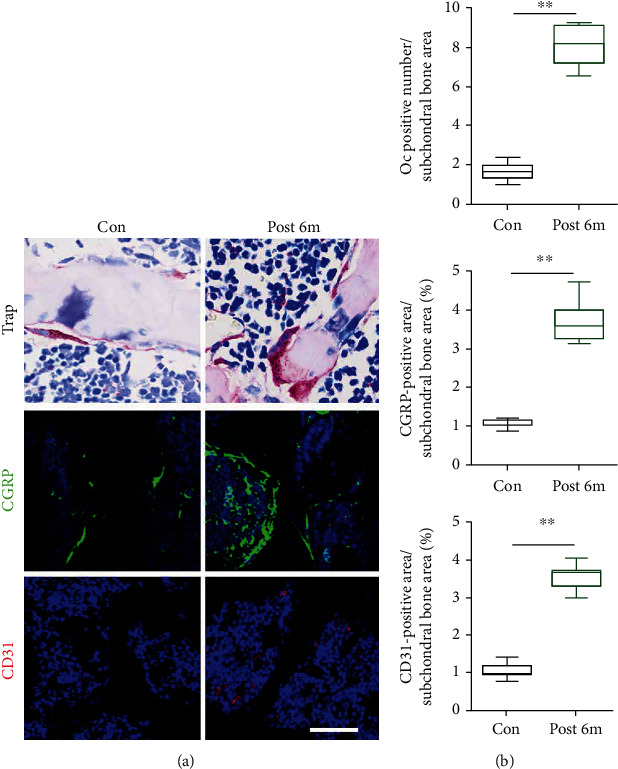
Mechanical loading accelerated subchondral bone resorption and aberrant vessel and nerve invade in the subchondral bone of LFJ. (a) Representative TRAP (upper), CGRP (middle), and CD-31- (lower-) positive blood vessel staining in LFJ were selected from different groups. (b) Quantitative analysis of osteoclast positive number, CGRP-positive areas, and CD31-positive areas in LFJ. The data are presented as the mean ± SD. ^∗∗^*p* < 0.01 for differences between the control group and the bipedal standing group. Scalebar = 40*μ*m.

Micro-CT data showed collapsed subchondral bone in the LFJ after long-term bipedal standing. We therefore explored the pathological changes with TRAP staining to visualize the osteoclast activity in the LFJ subchondral bone (Figure 5(a)). Trap staining revealed an increased number of osteoclasts (OC) in the LFJ OA mice (2 ± 1.2) compared to the control mice (8 ± 1.8) (Figure 5(b)). In addition, compared with the sham group, CD-31-positive vessels and GCRP-positive nerve endings increased significantly in LFJ OA mice (Figures 5(a) and 5(b)). However, the exact mechanism underlying the potential contributions of aberrant nerve invasion in the subchondral bone during LFJ osteoarthritis progression is largely unknown. Previous results demonstrate that long-term standing lead mice develop spine hyperalgesia. The spine pain maybe arises from the aberrant nerve invasion in the subchondral bone of LFJ.

## 4. Discussion

Lumbar spinal facet joint arthritis is considered clinically important sources of low back pain [19]. Animal models of LFJ OA are used extensively in research of its pathogenesis [20, 21]. Human beings are bipedal, and the loading acts on the lumbar spine were often assumed to be different from those in quadrupeds [22]. Thus, the biomechanical microenvironment of the lumbar spinal segments in humans is not the case in mice [17]. Therefore, as an essential step in the effort to explore the pathogenesis associated with facet joint degeneration, it is necessary to establish an animal model for properly representing human natural OA of LFJ.

Recently, research focusing on LFJ OA has been conducted using various animal models [2, 21]. In our current study, we reproduced a natural LFJ OA in a novel bipedal standing mouse model that was consistent with a previously described model [17]. Such an animal model is completely different from the chemically induced LF OA models, which creates a chemical injury to trigger LF OA and cannot stimulate the real pathological processes involved in human LFJ OA. Mechanical loading within a physiological range is necessary to maintain the spine in a healthy state [14, 23, 24]. LFJ is exposed to surprisingly large mechanical loads during standing movement. With lying or standing forces at the LFJ surface may vary from near zero to several times the whole-body weight within a period of 1 second [12, 25].

Although mechanotransduction can maintain tissue homeostasis in the joint, this process can also lead to tissue degeneration [26]. After increased long-term axial load stress on the LFJ in the bipedal standing mice, the aberrant mechanical loading act on the LFJ surface leads to cartilage degeneration, loss of extracellular matrix, and a decrease in proteoglycan, causing LFJ OA [17]. Subchondral bone changes in bone turnover, mineralization, and volume result in altered apparent are the typical hallmarks in the large knee joint OA development [27–30]. For the first time, to our knowledge, we characterized the histopathology feature change in the subchondral bone of LFJ OA after the long-term bipedal standing posture in mice. Micro-CT vividly demonstrated that the aberrant bone remodeling occurs in the subchondral bone during LFJ progression. Specifically, elevated osteoclast activity was found in the subchondral bone of LFJ accompanied by increased new blood vessel growth and aberrant nerve invasion. These results indicate that the aberrant nerve and vessel growth in the subchondral bone, after long bipedal standing, could be an important origin of LFJ OA pain and laid the important pathogenetic basis for the development of low back pain caused by LFJ OA. The axis mechanical loading act on the LFJ could activate nerve endings and modulate the signals in the nervous system to initiate the development of OA pain.

In animal models, the inhibition of the subchondral bone remodeling with pharmacological agents has demonstrated efficacy in the treatment of OA [28, 31, 32]. Our findings also suggest that the subchondral bone could be a therapeutic target for the management of LFJ OA pain. The increased remodeling rate in the subchondral bone of LFJ OA may be initiated by the excessive axial loading on the surface of LFJ, leading to the activation of the osteoclast activity [28, 33]. However, whether the aberrant nerve invasion and angiogenesis in the subchondral bone of LFJ OA were induced by osteoclasts is still unclear. How the mechanical loading signals acted on LFJ are converted into chemical information and leading activation of downstream signaling cascades in osteoclasts has rarely been explored. Osteoclast lineage cells are essential for bone remodeling and play an important role in maintaining bone homeostasis [34].

Beyond resorption, studies reveal several unanticipated roles for osteoclasts, which could secrete multiple factors, such as cytokines (clastokines) and growth factors, in the regulation of the bone remodeling cycle in health and disease status [35, 36]. A study showed that preosteoclasts secrete platelet-derived growth factor-BB (PDGF-BB) to induce angiogenesis coupled with osteogenesis during the bone remodeling process [37]. OA progression promotes both nerve and vessel growth in the osteoarthritic subchondral bone in the knee joint, leading to OA pain [33].

Other mechanisms may also exist for the osteoclast activation mediating pain. In cancer-associated bone pain (CABP), osteoclasts create an acidic extracellular microenvironment by secreting protons, which activate acid-sensing nociceptors and contribute to bone pain [38]. In ovariectomize (OVX) mice, two main classes, acid-sensing nociceptors, the transient receptor potential channel vanilloid subfamily member 1 (TRPV1), and acid-sensing ion channels (ASICs), are expressed in the sensory neurons innervating the bone and elicit pain signals when activated by acid stimuli related to the osteoclast activation during bone resorption [39]. Thus, the development of a specific osteoclast inhibitor targeting osteoclasts in the aberrant subchondral bone remodeling in LFJ OA may have effective pharmacological treatments that slow or halt disease progression and alleviating pain.

It would also be meaningful in future studies to examine the secrete factors by osteoclasts in the subchondral bone in a bipedal standing-induced LFJ OA model. In addition, mouse models offer the opportunity for genetic modification, and the corresponding genetically modified mice need to determine the main factor release by osteoclast-induced innervation in the subchondral bone in response to axial loading added on the LFJ [33].

The LFJ is a complicated biomechanical structure in the spine and has a complex mechanical performance [12]. Recently, there has been a growing interest in exploring the biomechanics and physiology of facet joints. Owing to the anatomical property of the spine, the mechanical behavior of the facet joint in each spinal segment is completely different [12, 40]. Thus, the axis mechanical stimulation act on the surface of each LFJ will initiate different intracellular signal cascade activations in various tissue components of the LFJ. This cascade includes the intracellular milieu (protein translation, gene transcription, posttranslational signaling) and intercellular signaling. However, this response has not been well defined in the subchondral bone of LFJ. The LFJ is formed by two adjacent vertebrae with the inferior articular process and superior articular process [12, 25]. The anatomy variations imply that the mechanical properties and cellular response vary within each part of the articular process. In our study, the superior articular process of the lumbar 5 segment was selected for systemic analysis. It will be interesting to further characterize the mechanical properties and physiology of LFJs among each segment.

## 5. Conclusion

Collectively, long-term axial loading induces the development of spine hyperalgesia in mice associate with increased osteoclast activity and aberrant angiogenesis and nerve invasion into the subchondral bone of LFJ that stimulate the natural pathological change in human LFJ OA. These results indicate that the aberrant bone remodeling associate with aberrant neve innervation in the subchondral bone has a potential as a therapeutic target in multiple LFJ OA pain.

## Data Availability

The datasets used and analyzed during the current study are available from the corresponding author on reasonable request.

## Abbreviations

OA: Osteoarthritis
LFJ: Lumbar facet joint
IDP: Intradiscal pressure
SAPs: Superior articular processes
OC: Osteoclast
OARSI: Osteoarthritis Research Society International
TRPV1: Transient receptor potential channel vanilloid subfamily member 1
ASICs: Acid-sensing ion channels
OVX: Ovariectomize.
